# *Salinarimonas chemoclinalis*, an Aerobic Anoxygenic Phototroph Isolated from a Saline, Sulfate-Rich Meromictic Lake

**DOI:** 10.3390/microorganisms12112359

**Published:** 2024-11-19

**Authors:** Katia Messner, John A. Kyndt, Vladimir Yurkov

**Affiliations:** 1 Department of Microbiology, University of Manitoba, Winnipeg, MB R3T 2N2, Canada; messner1@myumanitoba.ca; 2 College of Science and Technology, Bellevue University, Bellevue, NE 68005, USA; jkyndt@bellevue.edu

**Keywords:** *Salinarimonas*, bacteriochlorophyll *a*, aerobic anoxygenic phototroph, polyhydroxyalkanoate, *Salinarimonadaceae*, chemocline, meromictic lake, Mahoney Lake

## Abstract

A pink-pigmented, ovoid-rod-shaped, Gram-negative bacterial strain ML10^T^ was previously isolated in a study of a meromictic lake in British Columbia, Canada. It produces bacteriochlorophyll *a*, which is incorporated into the reaction center and light harvesting I complexes. This alongside no anaerobic or photoautotrophic growth supports the designation of the strain as an aerobic anoxygenic phototroph. The cells produce wavy polar flagellum and accumulate clear, refractive granules, presumed to be polyhydroxyalkanoate. Sequence of the 16S rRNA gene identified close relatedness to *Salinarimonas rosea* (97.85%), *Salinarimonas ramus* (97.92%) and *Saliniramus fredricksonii* (94.61%). The DNA G + C content was 72.06 mol %. Differences in cellular fatty acids and some physiological tests compared to *Salinarimonadaceae* members, as well as average nucleotide identity and digital DNA-DNA hybridization, define the strain as a new species in *Salinarimonas*. Therefore, we propose that ML10^T^ (=NCIMB 15586^T^ = DSM 118510^T^) be classified as the type strain of a new species in the genus with the name *Salinarimonas chemoclinalis* sp. nov.

## 1. Introduction

Mahoney Lake is located in south-central British Columbia, Canada. Originally named as such in 1936 [1], it was first examined for its properties in 1969. It has a surface area of 18 ha, depth of 18 m and is located 470 m above sea level [2]. There is no water in- or out-flow with levels primarily regulated through precipitation and evaporation [2]. The lake is meromictic, containing two water layers that do not mix: the bottom monimolimnion and the top mixolimnion. They are separated by a sharp chemical density gradient called the chemocline. This lake is commonly characterized as euxinic because it is both anoxic and sulfidic in the bottom layer [1] and is concentrated in dissolved solids (up to 85,000 mg/L) [2]. In contrast, the mixolimnion is oxygenated with a gradual decrease in levels until 8 m in depth at which oxygen is not present [3] and contains significantly less dissolved solids (10,000 mg/L) [2]. The saline waters of the lake are uniquely rich in MgSO_4_ compared to marine environments that are primarily dominated by NaCl [4]. Mahoney Lake became of great interest to microbiologists with the discovery that purple sulfur bacteria constitute most of the primary productivity [5], specifically, *Thiohalocapsa* sp. ML1 [6]. As such, studies on the microbial community continued to take place [3,7,8,9,10,11,12,13], which has resulted in the taxonomic characterization of new species and genera [12,13,14,15]. One bacterial group studied in the mixolimnion of the lake was aerobic anoxygenic phototrophs (AAPs) [3]. In total, 33 groups of AAPs and purple non-sulfur bacteria were isolated and characterized [3].

AAPs are bacteria that require oxygen not only for respiration, but also to conduct anoxygenic photosynthesis, a system primarily used anaerobically by other anoxygenic phototrophs. In addition, they cannot produce RuBisCo to survive autotrophically by fixing CO_2_ [16]. The energy derived from harvesting light allows them to grow competitively alongside other heterotrophs and survive low nutrient conditions. These core features differentiate the group, which primarily spans the α-, β- and γ-*Proteobacteria*.

*Salinarimonadaceae* is a relatively new family in *Hyphomicrobiales* [17]. Currently, the family contains four species, *Salinarimonas rosea*, *Salinarimonas ramus*, *Salinarimonas soli* and *Saliniramus fredricksonii* [18]. Most species have been isolated from saline environments including a salt mine [19], soil [20] and a lake [17]. The exception is *Salinarimonas soli*, which came from soil in South Korea and contrastingly has a very low salt tolerance, only growing in media containing up to 0.5% (*w*/*v*) NaCl [21]. The genus *Salinarimonas* is a group of Gram-negative, rod-shaped bacteria that are facultative anaerobic or obligate aerobic, motile, produce pink colonies, are catalase positive and contain Q-10 as the primary ubiquinone. The major cellular polar lipids are diphosphatidylglycerol, phosphatidylglycerol, phosphatidylmethylethanolamine and phosphatidylcholine [19]. Photosynthesis had been alluded to possibly be present in the genus with the discovery that they all contained genes for the photosynthetic reaction center (RC) [20,21]. However, only *Salinarimonas ramus* had been physically tested, with pigment extracts lacking bacteriochlorophyll *a* (Bchl *a*), possibly due to the conditions not being suited for photoheterotrophic growth [20]. Here, we describe a new AAP isolated from the mixolimnion of a meromictic lake, which produces Bchl *a* and forms an RC and light-harvesting I (LHI) complex. Strain ML10^T^ represents a new species of the genus *Salinarimonas* for which the name *Salinarimonas chemoclinalis* is proposed.

## 2. Materials and Methods

### 2.1. Cultivation

ML10^T^ was isolated on medium N2 from a sample collected 3 m below the surface of Mahoney Lake (49°17′ N, 119°35′ W) [3]. For subsequent characterization, it was grown for 7 days in the dark at 28 °C on a shaking incubator, unless otherwise indicated. For long-term storage, cells were cryopreserved at −75 °C in modified N2 with 10% (*w*/*v*) organics and 30% (*v*/*v*) glycerol.

### 2.2. Pigment Analysis

Photosynthetic complexes from whole cells and extracted Bchl *a* were detected using absorbance spectra in the 300–1100 nm region with the Hitachi U-2010 spectrophotometer (Tokyo, Japan) [16]. For whole cell reads, ML10^T^ from N2 plates was suspended in 0.3 mL 20 mM TRIS-HCl buffer (pH 7.8) and 0.7 mL glycerol to minimize light scattering [12]. Pigments from cells grown under the same condition were extracted using acetone/methanol (7:2, *v*:*v*) [16].

### 2.3. Morphology and Physiology Tests

Cell shape and size were determined after 7 days of growth and motility after 1 day via phase contrast microscopy (Zeiss Axioskop 2, Jena, Germany). Gram staining [22] was performed alongside a KOH hydrolysis [23] for confirmation. Colony morphology was evaluated after 7 days of growth. The microscopy of negative-stained cells and TEM of ultra-thin sections were completed previously [3].

All liquid culture physiological tests were inoculated with 5% (*v*/*v*) of starter ML10 cells. Temperature optimum and range was evaluated at the following (°C): 4, 8, 12, 16, 20, 24, 28, 32, 37 and 42. Growth at a pH of 5.0 to 12.5 with 0.5 increments as well as NaCl and NaSO_4_ % (*w*/*v*) ranging from 0 to 20% at 1% intervals were studied. The utilization of complex (bactopeptone, casamino acids and yeast extract) and single carbon sources such as organic Na salts (acetate, butyrate, citrate, formate, glutamate, malate, pyruvate, succinate), sugars (fructose, glucose, lactose) and simple alcohols (ethanol and methanol) were determined at 0.5% concentration in liquid N2 cultures modified to exclude any other organics. Oxidase, catalase, nitrate reduction and indole tests, as well as hydrolysis of between 20, 40, 60 and 80, starch, gelatin and agar, were assessed [24]. Antibiotic susceptibility was evaluated using disk diffusion for the following (μg): ampicillin (10), chloramphenicol (30), erythromycin (15), imipenem (10), kanamycin (30), penicillin G (10 IU), polymyxin B (300 IU), nalidixic acid (30), streptomycin (10), and tetracycline (30). If no inhibition of growth was observed, ML10^T^ was considered resistant.

Photo- and chemo-heterotrophic anaerobic growth was checked in screw-capped tubes incubated in light and dark with liquid N2 and purple non-sulfur bacteria media (PNSb) [25]. The latter was supplemented with 0.3 g/L Na-Succinate [26] as an additional organic electron donor and 1.5% NaCl to match the salinity of N2. Further anaerobic growth testing was assessed with different inorganic electron donors by substituting the amino acids (cysteine and methionine) in PNSb with Na_2_S (1 mM) or Na_2_S_2_O_3_ (1 mM) [14]. The anaerobic fermentation of individual carbon sources was performed in the dark using screw-capped tubes filled with liquid minimal N2 and the following sugars (0.5% *w*/*v*): glucose, fructose, lactose maltose and sucrose. Durham vials were added to determine if CO_2_ was produced. Aerobic photoautotrophy was evaluated in liquid, organic-free PNSb, supplemented with (g/L) 1.5 NaHCO_3_ and 0.5 Na_2_S_2_O_3_ as an inorganic carbon and sulfur source/electron donor, respectively [27], and grown in the presence of an incandescent light bulb (2270 lx, measured with a light meter). Two subsequent cell transfers were conducted if growth occurred to account for organics that remained in the initial inoculum.

### 2.4. Chemotaxonomy

ML10^T^ was grown on N2 plates for 3 days at 28 °C in the dark. Cells were collected in triplicate, lipid extractions and purification were performed using Folch’s method [28], then analyzed with gas chromatography to identify fatty acid composition [29].

### 2.5. 16S rRNA and Genomic Sequencing

DNA was extracted and the partial 16S rRNA gene was Sanger sequenced with universal primers 27F (5′-AGAGTTTGATCCTGGCTCAG-3′) and 1492R (5′-GGTTACCTTGTTACGACTT-3′). Chromatograms were processed on DNA Baser Assembler v4.36.0 (Heracle BioSoft SRL, Mioveni, Romania). Most related type species for ML10^T^ based on this gene was identified through a standard nucleotide BLAST search [30]. A 16S rRNA phylogenetic tree was constructed with MEGA X software [31], using the Maximum Likelihood method and 1000 bootstrap replicates. The evolutionary history was inferred with the Maximum Likelihood method. The Tamura three-parameter model [32] selected for the tree was determined with the MEGA X ‘find best DNA model’ tool. To measure evolutionary rate differences among sites, a gamma distribution (+G, parameter = 0.5022) with five rate categories was applied and it was assumed a certain fraction of sites are evolutionarily invariable ([+I], 35.61% sites). The final dataset had 36 nucleotide sequences and 1522 positions.

To obtain the whole genome sequence, extracted DNA was prepared using the Illumina DNA Library Prep Kit (San Diego, CA, USA). From this, 500 µL of 1.8 pM was paired-end (2 × 150 bp) sequenced in the Illumina MiniSeq system. This generated 862.27 Mbp comprising 5,710,366 reads. The dataset was quality checked with FASTQC (version 1.0.0) using a k-mer size of 5 and contamination filtering for overrepresented sequences against the default contamination list. FASTQ Toolkit (version 2.2.6) was applied to clean the sequences. This included the trimming of Ns from the 3′ end before identifying adapters; PCR common sequence; bases at 3′ and 5′ ends with a Q score < 30. The finalized reads were assembled de novo using Unicycler (version 0.5.0) [33] through the BV-BRC Comprehensive Genome Analysis tool [34]. G + C content (mol %) was determined using the genome sequence. Annotation was completed with the Prokaryotic Genome Annotation Pipeline [35]. A phylogenetic tree based on 467 single-copy PATRIC global protein family (PGFam) genes found in 24 genomes of ML10^T^’s closely related species within Hyphomicrobiales was constructed using RAxML (version 8.2.11) within the BV-BRC Bacterial Genome Tree Tool [34,36]. The program’s Fast Bootstrapping was run for branch support (100 rounds).

Digital DNA-DNA hybridization (dDDH) and confidence intervals were calculated with the Genome-to-Genome Distance Calculator (v 4.0) applying the recommended settings [37,38] on the Type (Strain) Genome Server (TYGS) [37]. Average nucleotide identity (ANI) was assessed with JSpeciesWS [39].

The 16S rRNA partial gene was deposited in GenBank under accession number PQ133587. This Whole Genome Shotgun project has been deposited at DDBJ/ENA/GenBank under the accession JBGMWH000000000 and is the version used in this work.

## 3. Results and Discussion

### 3.1. Spectral Analysis

**Figure 1:**
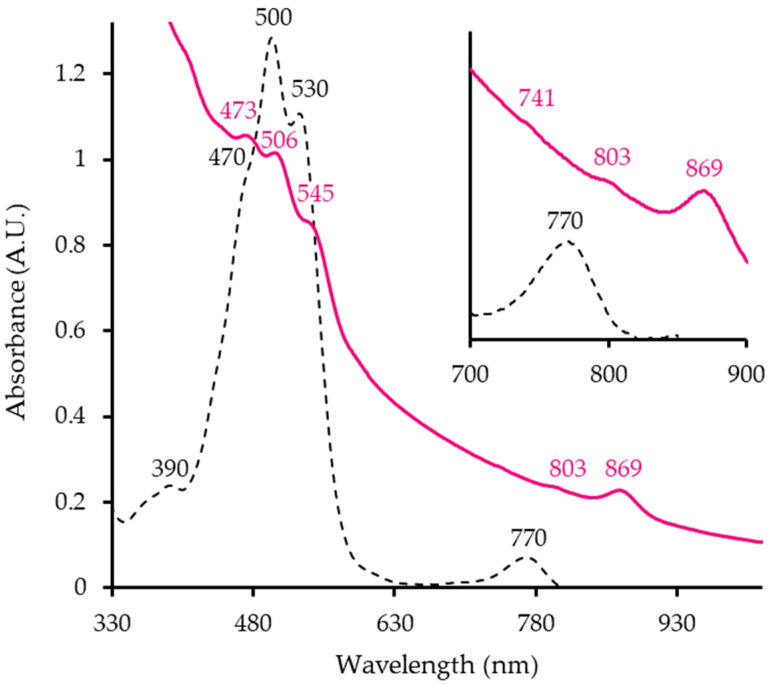
Whole cell (pink line) and pigment extract (black, dashed line) absorbance spectra of ML10^T^. Values of importance are indicated for each spectrum with the respective color of the line.

The absorbance spectrum of ML10^T^ whole cells shows typical peaks of an anoxygenic photosynthetic apparatus (Figure 1). The strain has an LHI complex, indicated at the 869 nm peak (Figure 1, pink line) as well as RC (803 nm, 741 nm). Hence, ML10^T^ has a membrane-bound photosynthetic-pigment complex. Peaks in the 400–550 nm range are responsible for cells’ pink hue. The pigment extract absorption spectrum (Figure 1 black, dashed line) shows that it is synthesizing Bchl *a* (770 nm), although at a much lower proportion relative to the carotenoids (470 nm, 500 nm, 530 nm). This ratio is reflective of AAP since only a small portion of secondary pigments is usually associated with LH complexes and the majority is found across the membrane, presumably helping the cell deal with oxidative stress [16]. These findings are valuable as it is the first evidence of photosynthesis taking place in *Salinarimonadaceae*. Based on 16S rRNA gene phylogeny, the closest known AAP relative to ML10^T^ is *Bosea lupini* [40]. Previously, no Bchl *a* was detected in the analysis of *Salinarimonas ramus* [20]. However, in past studies, genes for the RC proteins were identified in the genomes of all *Salinarimonas* spp., [20,21]. Therefore, it is possible that *Salinarimonas ramus* and other members can use light to produce chemical energy (ATP) for cell activities.

### 3.2. Culture and Cell Features

**Figure 2:**
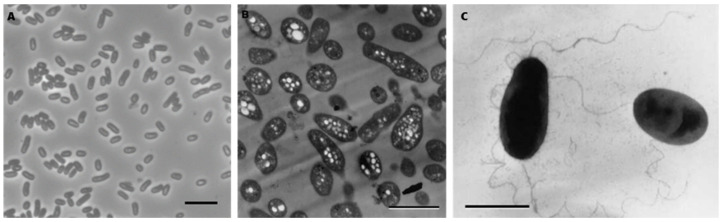
Cellular morphology of ML10^T^. (**A**) Phase-contrast image; (**B**) Electron micrograph showing extensive accumulation of polyhydroxyalkanoate granules [3]; (**C**) negatively stained cells, highlighting single and possibly multiple polar wavy flagellation [3]. Bars are 5 μm (**A**), 2 μm (**B**) and 1 μm (**C**).

When grown aerobically on N2 for 7 days, ML10^T^ forms raised, convex, circular, glossy punctiform colonies with a soft texture. Like most members of *Salinarimonadaceae*, they have a pink appearance. It is gram-negative, confirmed with staining, KOH test and electron microscopy [3]. Cells are ovoid rods (Figure 2A) and after 7 days of growth are 2.1 ± 0.4 µm in length and 1.0 ± 0.1 µm in width. In electron micrographs, it is shown that they accumulate clear intracellular granules, potentially containing polyhydroxyalkanotae(s) (PHA) (Figure 2B) [3]. Similar compounds have also been detected in *Salinarimonas rosea* and *Salinarimonas ramus* [20]. Furthermore, PHA accumulation is commonly found in AAP, with production depending on light availability and carbon source [41]. They are motile, powered by single or possibly multiple polar wavy flagella (Figure 2C) [3].

### 3.3. Physiology and Biochemistry

ML10^T^ was isolated from a saline environment like *Salinarimonas rosea* and *Salinarimonas ramus*. As such, the NaCl range and optimum of the strain is similar to those members (Table 1) and significantly different from *Salinarimonas soli*. Mahoney Lake is sulfate-rich; therefore, the Na_2_SO_4_ tolerance was also determined. The strain could grow from 0 to 9%, with the optimum being at 8%. The ideal temperature for mesophilic ML10^T^ is the highest among the genus at 37 °C, with the others preferring around 28–30 °C (Table 1). It also has a greater range of alkali tolerance and lower optimal pH. ML10^T^ could neither survive in any of the anoxic conditions tested, including fermentation of sugars, nor could it grow photoautotrophically. For this reason, it is classified as an AAP, since it demonstrates the core features: Bchl *a* production, oxygen requirement and photoheterotrophy. Salinarimonas soli is also an obligate aerobe; however, most related *Salinarimonas rosea* and *Salinarimonas ramus* are facultative anaerobes (Table 1).

ML10^T^ was positive for oxidase, catalase and gelatinase, the latter of which has not been observed in *Salinarimonas*. It could not hydrolyze tweens, and was negative for indole, amylase and nitrate reductase. As most AAPs, the strain can use a variety of carbon sources including acetate, pyruvate, glutamate, malate, succinate, lactate, glucose and yeast extract. It is susceptible to all antibiotics tested except nalidixic acid and polymyxin B. It requires the vitamins provided for growth. The utilization of these carbon source under the minimal medium conditions also confirmed that ML10^T^ could use ammonium and sulfate as a N and S source, respectively, since these were the only ones provided under these conditions and nitrogen fixation is not possible as these genes are not present in the genome.

Fatty acid analysis highlighted differences in the lipid composition of ML10^T^ and other *Salinarimonadaceae* (Table 2). It shared its most prevalent fatty acid, C_18:1_ ω7c with all *Salinarimonas* spp. as well as C_16:0_ and C_18:0_. Alternatively, it did not have C_14:0_ 3-OH/iso-C_16:1_, C_16:1_ ω7c/C_16:1_ ω6c, C_19:0_ cyclo ω8c or C_20:1_ ω7c as the other members of the genus did. The strain had C_18:1_, C_16:1_ in common with only *Saliniramus fredricksonii*, although in different proportions. C_17:0_, C_20:4_, C_20:5_ are exclusive to ML10^T^.

### 3.4. Genome Characteristics and Phylogeny

**Figure 3:**
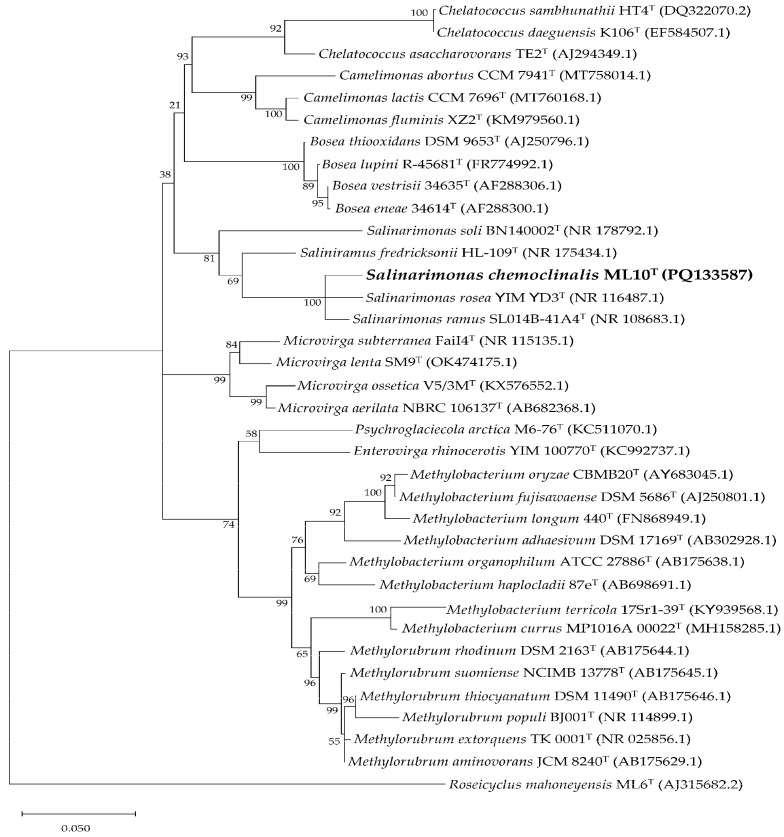
Phylogenetic tree of ML10^T^ and strains from most related genera based on 16S rRNA gene sequences. Created using maximum likelihood method with ×1000 bootstraps. Version with highest log likelihood (−8210.23) is presented and drawn to scale. Branch lengths measured in number of substitutions per site. The percentage of trees in which the associated taxa clustered together is shown next to the branches. Accession number for sequences used included in parentheses.

According to standard nucleotide BLAST results of the partial 16S rRNA gene sequence of ML10^T^ (1398 bp), the most related species were *Salinarimonas rosea* (97.92%), *Salinarimonas ramus* (97.85%) *Saliniramus fredricksonii* (94.61%). This aligned with the observed similarities in their physiology. Including these and other 16S rRNA fragments from closely related genera, a maximum likelihood tree was constructed (Figure 3). Interestingly, *Saliniramus fredricksonii* more closely related to ML10^T^ than the other member of the genus, *Salinarimonas soli*.

ML10^T^’s genome is 4,753,738 bp, making it slightly smaller than the other *Salinarimonas* (Table 3). It contained 42 contigs and a 177X average fold coverage. No plasmids were found. The G + C content at 72.06 mol % is higher than the others, but still within reasonable range for the genus. The full 16S rRNA sequence was identified in the genome (1491 bp). A total of 4629 coding sequences were identified, matching expected outputs for this genome size. ML10^T^ had 89 annotated protein sequences, which are absent in the other *Salinarimonadaceae* genomes. The majority were hypothetical; however, the CRISPR-associated Cas9 and Cas2 as well as phage-related genes were present. This suggests ML10^T^ may have the capability to combat bacteriophage infections with the CRISPR system. Like other AAP and *Salinarimonas* members, it did not encode RuBisCo [42]. As observed in the absorbance spectra, (Figure 1), it also contained all the genes required for anoxygenic photosynthesis. Analysis of the other species in the genus showed they also have these genes, increasing the likelihood that they can also harness light energy for ATP production. Definitive proof is still needed, especially as *Salinarimonas ramus* and *Salinarimonas rosea* are facultative anaerobes and their oxygen requirement for the expression of anoxygenic photosynthesis would affect whether they are characterized as a new type of AAP or as a purple non-sulfur bacteria. Although *Saliniramus fredricksonii* was reported to have no photosynthetic genes [17], it encodes a xanthorhodopsin and therefore potentially utilizes light energy. PHA synthase and polyhydroxybutyrate (PHB) depolymerase sequences in ML10^T^’s genome supports the conclusion that the intracellular granules accumulated in cells are a PHA.

The ANI for ML10^T^ alongside all species in the family is lower than 95% [42] and dDDH is below the 70% cut-off for species designation (Table 4) [38,43].

**Figure 4:**
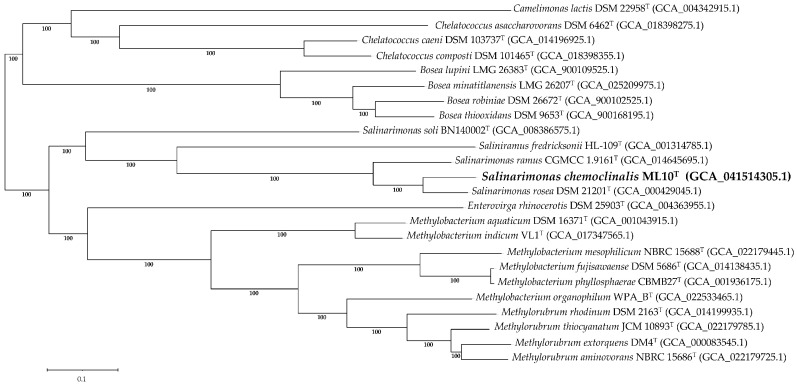
Phylogenetic tree of multi-loci sequence alignment of 467 genes from PGFams identified within the selected group of genomes in *Hyphomicrobiales*. The branch support values were generated from 100 rounds of ‘Rapid bootstrapping’ in RaxML. Tree scale was defined as the mean number of substitutions per site, averaging both nucleotide and amino acid changes. GenBank assembly accession number is included in parenthesis.

Similar to the 16S rRNA tree, a genome-based phylogenetic tree found that ML10^T^ formed a distinct lineage among the *Salinarimonadaceae* (Figure 4), supporting classification of the strain as a new species. *Salinarimonas soli* was also the most divergent from the other family members despite *Saliniramus fredricksonii* residing in a different genus. To resolve this contradiction, a separate study with further analysis of the relationship between the species in *Salinarimonadaceae* must be conducted.

## 4. Conclusions

Polyphasic analysis of ML10^T^ supports its classification as a new species within *Salinarimonas*. As such, we propose the new name *Salinarimonas chemoclinalis*, with ML10^T^ as the type strain.

### 4.1. Emended Description of Salinarimonas Genus (Liu et al. 2010) [19]

It is as published by Liu et al. [19] with the following amendments. Genus includes facultative anaerobes as well as obligate aerobes. All members contain genes for bacteriochlorophyll *a*, carotenoid and light-harvesting complex synthesis required for anoxygenic photosynthesis as well as for polyhydroxyalkanoate intracellular accumulation.

### 4.2. Description of Salinarimonas chemoclinalis sp. Nov.

*Salinarimonas chemoclinalis* (che.mo.cli.na’lis. N.L. fem. adj. chemoclinalis referring to chemocline zone in meromictic Mahoney Lake).

Cells are ovoid, motile with a single or possibly multiple polar flagella and are approximately 2.1 ± 0.4 µm in length and 1.0 ± 0.1 µm in width after 7 days of growth. Gram-negative, oxidase-positive, catalase-negative. Grows in the following conditions (optimum): temperature range of 12 to 41 °C (37 °C); pH of 6.0 to 10.0 (6.5); NaCl (% *w*/*v*) at 0 to 10% (6%); and NaSO4 (% *w*/*v*) at 0 to 9% (8%). After growing for 7 days on N2 medium, colonies are pink, raised, convex, circular, glossy and punctiform with a soft texture. Bacteriochlorophyll *a*, carotenoids, photosynthetic reaction center and light-harvesting I complex are produced, indicative of aerobic anoxygenic photosynthesis. It does not have nitrate reductase, amylase or tryptophanase. It can hydrolyze gelatin, but not between 20, 40, 60 or 80. It is able to use acetate, pyruvate, glutamate, malate, succinate, lactate, glucose and yeast extract, but not butyrate, citrate, formate, fructose, lactose, methanol, ethanol, bactopeptone or casamino acids as carbon sources. Cells are sensitive to (μg) ampicillin (10), chloramphenicol (30), erythromycin (15), imipenem (10), kanamycin (30), penicillin G (10 IU), streptomycin (10) and tetracycline (30) but resistant to polymyxin B (300 IU) and nalidixic acid (30). Major fatty acids (>4%) are C_18:1_ ω7c, C_16:1_, C_16:0_ and minor fatty acids are C_14:0_, C_17:0_, C_18:0_, C_18:1_, C_20:4_ and C_20:5_. The genome is 4.75 Mbp and has a G+C content of 72.06 mol %.

The type strain is ML10^T^ (=NCIMB 15586^T^ = DSM 118510^T^). It was isolated from Mahoney Lake, a saline- and sulfate-rich meromictic lake in British Columbia, Canada. The GenBank accession numbers for the 16S rRNA gene sequence and genome assembly of ML10T are PQ133587 and GCA_041514305.1, respectively.

## Figures and Tables

**Table 1:** Differential characteristics of ML10^T^ from other *Salinarimonas* species [19,20,21].

Species	*Salinarimonas chemoclinalis*	*Salinarimonas rosea*	*Salinarimonas ramus*	*Salinarimonas soli*
Strain	ML10^T^	YIM YD3^T^	SL014B-41A4^T^	BN140002^T^
Habitat	Meromictic lake	Salt mine	Crude oil-polluted saline soil	Soil sample
Location	British Columbia, Canada	Yunnan, China	Eastern China	Goesan-gun, Republic of Korea
Colony colour	Pink	Pink	Deep red	Light pink
Genomic evidence of photosynthesis	+	+	+	+
Cell shape	Ovoid to rod, occasionally elongated	Rods	Straight or curved rods, usually forming branches	Rods
Cell size (µm)	2.1 ± 0.4 × 1.0 ± 0.1	0.4–1.0 × 1.20–1.45	0.6–4.0 × 1.25–25	0.8–1.0 × 1.4–1.6
Motility	+	+	+	+
Temperature range/optimum (°C)	12–41/37	15–37/28–30	4–50/28	15–37/25–30
pH range/optimum	6.0–10.0/6.5	6.0–9.0/7.0–8.0	6.0–9.0/7.0	7.0–9.0/8.0
NaCl range/optimum (*w*/*v*, %)	0–10/6	0–5/3	0–10/4	0–0.5/ND
Oxygen requirement	Obligate aerobe	Facultative anaerobe	Facultative anaerobe	Obligate aerobe

+, Positive.

**Table 2:** Cellular fatty acid composition of ML10 compared to related members of the *Salinarimonadaceae* family [17,19,20,21].

Species	*Salinarimonas chemoclinalis*	*Salinarimonas rosea*	*Salinarimonas ramus*	*Salinarimonas soli*	*Salinariramus fredricksonii*
Strain	ML10^T^	YIM YD3^T^	SL014B-41A4^T^	BN140002^T^	HL-109^T^
Fatty Acid (%) *					
C_12:0_ aldehyde and/or unknown 10.98	-	-	-	1.1	-
C_14:0_ 3-OH/iso-C_16:1_	-	<1	1.2	1.7	-
C_14:0_	<1	-	<1	-	<1
C_14:0_ 3-OH	-	-	-	-	<1
C_16:0_	4.0	4.5	7.7	<1	15.1
C_16:0_ 3-OH	-	-	-	-	<1
C_16:1_	5.6	-	-	-	2.0
C_16:1_ ω5c	-	-	-	2.7	-
C_16:1_ ω7c/C_16:1_ ω6c	-	3.10	<1	<1	-
C_17:0_	<1	-	-	-	-
C_17:0_ cyclo	-	<1	-	-	-
C_18:0_ 3-OH	-	<1	<1	1.1	<1
C_18:0_ cyclo	-	-	-	-	9.7
C_18:0_	3.8	4.9	8.8	1.4	22.5
C_18:1_	<1	-	-	-	46.8
C_18:1_ ω7c and/or C_18:1_ ω6c.	-	71.1	73.8	84.2	-
C_18:1_ ω7c 11-methyl	-	3.0	2.5	-	-
C_18:1_ ω7c	85.5	-	-	-	-
C_19:0_	-	-	-	-	<1
C_19:0_ cyclo ω8c	-	7.2	1.6	3.3	-
C_20:1_ ω7c	-	3.7	2.1	1.2	-
C_20:4_	<1	-	-	-	-
C_20:5_	<1	-	-	-	-
C_21:1_	-	-	-	-	<1
C_22:1_	-	-	-	-	<1

-, not detected. * Values rounded to nearest tenth place.

**Table 3:** Genomic features in *Salinarimonas* species.

Species	*Salinarimonas chemoclinalis*	*Salinarimonas rosea*	*Salinarimonas ramus*	*Salinarimonas soli*
Strain	ML10^T^	YIM YD3^T^	SL014B-41A4^T^	BN140002^T^
GenBank assembly Accession number	GCA_041514305.1	GCA_000429045.1	GCA_014645695.1	GCA_008386575.1
Genome Size (Mb)	4,753,738	5,244,776	5,122,378	5,353,455
Mean sequencing coverage (fold)	177	ND	142	46
Completeness (%)	100	100	100	99.31
No. of contigs	42	62	54	292
L50	5	9	6	13
N50	234,929	220,202	304,224	142,509
G + C content (mol %) ^1^	72.06	71.80	70.58	70.07
No. of protein-coding sequences	4640	4952	5004	5578
No. of rRNA operons	3	5	4	3
No. of tRNA operons	46	43	57	46

ND, no data. ^1^ Determined using genome sequence.

**Table 4:** ANI, dDDH and confidence interval (C.I) values of reference genome ML10^T^ and *Salinarimonadaceae* members.

Species		*Salinarimonas rosea*	*Salinarimonas ramus*	*Salinarimonas soli*	*Salinramus fredricksonii*
Strain		YIM YD3^T^	SL014B-41A4^T^	BN140002^T^	HL-109^T^
ANI		89.0	84.1	74.3	73.3
Formula (1)	dDDH (%)	61.1	50.1	16.2	15.2
	C.I. (%)	57.4–64.6	46.7–53.5	13.2–19.7	12.3–18.6
Formula (2)	dDDH (%)	38.4	27.7	20.7	20.3
	C.I. (%)	35.9–40.9	25.3–30.2	18.5–23.1	18.0–22.7
Formula (3)	dDDH (%)	56.0	43.2	16.1	15.3
	C.I. (%)	52.8–59.1	40.2–46.2	13.6–19.1	12.8–18.1

## Data Availability

The ML10^T^ 16S rRNA sequence has been deposited in GenBank with the accession number PQ133587. This Whole Genome Shotgun project has been deposited at DDBJ/ENA/GenBank under the accession JBGMWH000000000, which is used in this work.
